# Improving the Utility, Safety, and Ethical Use of a Passive Mood-Tracking App for People With Bipolar Disorder Using Coproduction: Qualitative Focus Group Study

**DOI:** 10.2196/65140

**Published:** 2025-02-07

**Authors:** Laurence Astill Wright, Matthew Moore, Stuart Reeves, Elvira Perez Vallejos, Richard Morriss

**Affiliations:** 1 Institute of Mental Health University of Nottingham Nottingham United Kingdom; 2 Centre for Academic Mental Health Population Health Sciences University of Bristol Bristol United Kingdom; 3 NIHR MindTech Medical Technology Collaborative University of Nottingham Nottingham United Kingdom; 4 School of Computer Science University of Nottingham Nottingham United Kingdom; 5 NIHR ARC East Midlands University of Nottingham Nottingham United Kingdom; 6 Nottingham NIHR Biomedical Research Centre University of Nottingham Nottingham United Kingdom

**Keywords:** mood monitoring, ecological momentary assessment, EMA, passive ecological momentary assessment, passive EMA, bipolar disorder, implementation, qualitative, mobile phone

## Abstract

**Background:**

Coproduction with users of new digital technology, such as passive mood monitoring, is likely to improve its utility, safety, and successful implementation via improved design and consideration of how such technology fits with their daily lives. Mood-monitoring interventions are commonly used by people with bipolar disorder (BD) and have promising potential for digitization using novel technological methods.

**Objective:**

This study aims to explore how a passive behavioral monitoring platform, Remote Assessment of Disease and Relapse, would meet the needs of people with BD by specifically considering purpose and function, diversity of need, personal preference, essential components and potential risks, and harms and mitigation strategies through an iterative coproduction process.

**Methods:**

A total of 17 people with BD were recruited via national charities. We conducted 3 web-based focus groups as a part of an iterative coproduction process in line with responsible research and innovation principles and with consideration of clinical challenges associated with BD. Data were analyzed thematically. Results were cross-checked by someone with lived experience of BD.

**Results:**

Focus groups were transcribed and analyzed using thematic analysis. Six themes were identified as follows: (1) the purpose of using the app, (2) desired features, (3) when to use the app, (4) risks of using the app, (5) sharing with family and friends, and (6) sharing with health care professionals.

**Conclusions:**

People with BD who are interested in using passive technology to monitor their mood wish to do so for a wide variety of purposes, identifying several preferences and potential risks. Principally, people with BD wished to use this novel technology to aid them in self-managing their BD with greater insight and a better understanding of potential triggers. We discuss key features that may aid this functionality and purpose, including crisis plans and sharing with others. Future development of passive mood-monitoring technologies should not assume that the involvement of formal mental health services is desired.

## Introduction

### Background

Recent developments in technology have increased the possibility of determining the mental state of an individual via tracking behavioral data collected via smartphones [1]. This promises novel interventions based on passive ecological momentary assessment—a technology that can potentially frequently assess mood via data collected in the background of one’s smartphone use [2]. While this hypothesis remains controversial, many research teams are trying to implement this new technology into usable and acceptable interventions [3]. One open-access research platform using passive monitoring technology to glean new insights into mental disorders is the Remote Assessment of Disease and Relapse (RADAR) [4]. Here, we explore the design and potential implementation of this technology, using the RADAR platform, in the form of a mood-tracking app for people with bipolar disorder (BD), considering purpose, function, components, and risks and harms in an iterative coproduction process centrally involving end users of the technology—people with BD interested in mood monitoring. However, clinical experts and experts in the design of digital technology involving human-computer interaction and responsible innovation were also involved in such coproduction, as we have previously proposed [5]. There is a tension between 2 models of technology explored here—one where mental state is predicted via smartphone use and another where technology enters our lives via coproduction. This paper builds on these conceptual ideas.

BD is a condition where individuals already apply various mood-tracking methods to try to gain insights into the disorder and prevent relapse [5,6]. There is some evidence suggesting that increasing awareness of mood fluctuations can improve insights into and the identification of early warning signs and can be useful in preventing relapse in BD [7]. It also suggests that novel technology increases the potential to harness this new technology to create more sophisticated and structured mood-tracking interventions for people with BD. Most of these existing techniques and methods of tracking mood require considerable effort from participants in logging their mood every day for many months [8,9] and may be subject to inaccurate information due to mood biases [10]. Many people with BD use approaches such as paper- and pen-based tracking procedures, mental and physical notation, conversations with their partner, or already existing active smartphone apps [5]. Passive data collection approaches (which are then interpreted actively by the user) may improve flexibility, usability, and acceptability and decrease the time requirement for people who monitor their moods. In a previous qualitative work, a passive mood-tracking app was perceived as potentially positive and helpful by people with BD [5], solving many of the issues with existing methods.

When applying novel technologies in potentially clinical and vulnerable populations, it is important to consider potential risks and harms [11,12]. Technical advances offer potential solutions to complex problems with lighter regulation than many existing approaches to mental health, for example, medication. There is an urgent need to embed responsible research and innovation (RRI) frameworks to maximize societal benefit and be able to anticipate and reflect on the medium- or long-term impact of technology on stakeholders as well as the environment [13]. For example, by engaging with users, developers can identify design properties that could minimize the potential for harm in a number of ways (eg, accessible interfaces, integration with conventional mental health services, and enhanced data privacy and security [14]) and also reduce other risks, for example, digital exclusion that might potentially increase inequalities [14-16].

RRI [11] provide an ethical approach that requires planning, anticipation, and co-design through technological development with end users and all those involved in developing and later using the technology. Along with mitigating harm and inequality, such a co-design process carried out in the framework helps to improve its acceptability and usability for all potential users and relevance to practice and provides some assurance of the safety of the product. For these reasons, a co-design process may lead to a faster spread of usable interventions and a quicker abandonment of less satisfactory developments. However, one element that has so far been given less consideration in the work of responsible innovation is the range of problems associated with different types of mental states when using different forms of technology, such as digital mood monitoring, particularly in conditions where change may be dramatically different, such as BD, where people with BD may experience more than one type of mental state, such as mania and depression. For instance, people with mania are likely to show dramatic changes in their social contacts and what they will communicate and share (particularly on social media) compared to when they are depressed or when they are in neither mental state. This paper applies principles from RRI to consider risk anticipation and risk mitigation in people with BD.

RADAR is an open-source platform and resource for self-tracking psychiatric and neurological conditions developed by a consortium of academic and industry organizations. Currently, the platform has been successfully developed for those with multiple sclerosis [17], depression [4], and epilepsy [18]. RADAR currently passively monitors the following smartphone data: phone use, local weather, step count, GPS location, app use and time duration, battery level, Bluetooth devices in the nearby vicinity, actigraphy, heart rate, call or SMS text message logs, and ambient light.

We are aiming to apply the technology to people with BD, to assess how it might be adapted and adjusted to meet the specific needs of this population. We will not only focus on the technology but also on the requirements needed to ensure it fulfills the aspirations of people with BD, balanced against identifying potential risks. We will use expertise in responsible technology design to consider unintended consequences, potential risks, and how these might be mitigated.

### Objectives

We conducted a series of focus groups to explore the opportunities and challenges of the app to ensure the acceptability and efficacy of passive mood-monitoring apps in the future. This work was part of an iterative coproduction process in line with the use of RRI. We aimed to determine opinions on the following: (1) the purpose and function of the app, (2) the essential and desired components of the digital tool and optimal methods of delivery and design frequency, and (3) the potential risks and harms of using the technology and how these might be mitigated

## Methods

### Overview

Participants were recruited via national and local charities (Bipolar UK and Bipolar LIFT CIC) via their research opportunity network and other snowballing techniques via an existing patient and public involvement (PPI) team based at the Institute of Mental Health and the University of Nottingham.

Previous research included 16 individuals with BD [5,19] in 2 workshops; however, this explored the utility of self-tracking in general before the development of the RADAR-based tool and did not explore the tool in detail. We chose to explore the study aims through 3 focus groups with potential end users of this technology to simulate discussion and exploration of these issues rather than conduct individual interviews. Previously, we recruited participants from a geographical area that was familiar with participation in research on BD and digital mental health. We wanted to check whether the findings would apply to participants who were not from an area where there was such exposure to research so that fresh ideas and challenges might emerge and also to check the transferability of previous findings. We wanted to test the credibility and confirmability by feeding back findings from previous workshops and focus groups. To facilitate this, one of the workshop leaders from our previous research (an individual with over a decade of experience in PPI and who also has experienced BD) took part in the development of the topic guides and write-up of the current project. All these approaches enhanced the trustworthiness of data collection and analysis. We hoped to recruit 5 to 8 individuals per focus group (>8 individuals would be inappropriate via video call). We planned to run as many focus groups as required until there was a saturation of themes emerging from the focus groups, and no new themes were emerging. Initially, we planned 3 focus groups based on our previous experience of carrying out workshops and focus groups.

The inclusion criteria were as follows: self-reported formal diagnosis of bipolar 1 disorder, bipolar 2 disorder, or bipolar spectrum disorder (BD not otherwise specified or cyclothymia) by a UK psychiatrist, owns and regularly uses a smartphone with an interest in self-tracking their mood via a digital tool, fluent in English, able to give informed consent, and aged >16 years. Exclusion criteria were as follows: participants who lacked the capacity to consent (eg, self-reported diagnosis of dementia and primary diagnosis of substance misuse) or were current inpatients in a psychiatric hospital.

The study was conducted between August and December 2023. We tried to ensure that our methods reflected current practices used by these organizations who organize workshops, focus groups, and group events, which was the reason behind our choice of data collection. Our 3 focus groups were held on the web to improve accessibility and to improve diversity recruitment. The COREQ (Consolidated Criteria for Reporting Qualitative Studies) checklist is presented in Multimedia Appendix 1.

### Ethical Considerations

The study was subject to ethical review and approved by the faculty of medicine and health sciences at the University of Nottingham (FMHS 174-1222). Participants made first contact with the research team following advertising. Participants were initially provided with a participant information sheet and given an opportunity to discuss this and ask questions via phone call or email. After ≥48 hours, participants were invited to complete the consent form and again provided with an opportunity to ask questions via telephone call or an alternative method. The data in this study have been deidentified where applicable, and participants consented to the publishing of deidentified quotations. Participants were provided with a £30 (US $38) voucher following the completion of a single focus group. The participants had no relationship with the research team before joining the study. The participants knew about the reasons for conducting the research but had limited knowledge about the research team other than their qualifications. While the research team was diverse, including people with lived experience, some members were psychiatrists and therefore might have carried certain assumptions or biases about the delivery of interventions in people with BD in the National Health Service.

### Focus Group Layout

**Figure 1:**
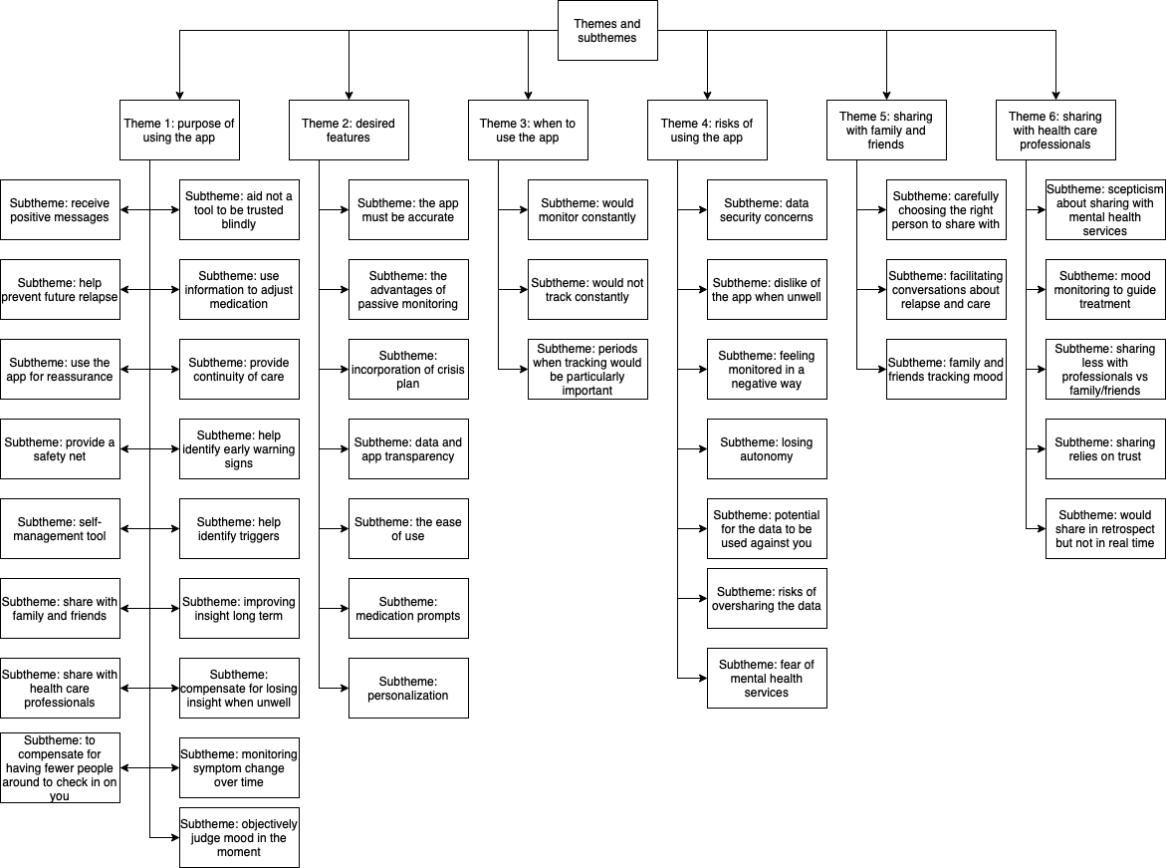
Overview of the main findings of the 6 themes.

Each focus group lasted 2 hours in total and followed a semistructured topic guide informed by previous research [5] and produced by 2 psychiatrists and 1 person with lived experience of BD (LAW, RM, and MM). Participants were given an explanation of the purpose of the focus groups before participation. The topic guide was used to ask open questions on (1) developing the function of the mood-tracking tool, (2) developing the content of the mood-tracking tool, and (3) how best to translate function and content into a usable and acceptable digital tool (Figure 1). The first 2 focus groups concentrated mostly on what participants wanted from the app and why, reaching saturation in relation to these themes. Therefore, the third focus group only briefly commented on these matters having been presented with these findings. The third focus group concentrated in more detail on how the app was delivered, although some of this content had been explored in the first 2 focus groups. Focus groups were video recorded followed by verbatim transcription. Focus groups were facilitated by LAW (male psychiatrist), who was trained in qualitative interviewing and focus group data collection. At the time of the focus group, all participants knew LAW from their first contact when considering joining the study.

Participants in all focus groups were given a verbal description of the RADAR platform, including the type and amount of data it was possible to collect presented pictorially (Figure S1 in Multimedia Appendix 2). Participants in focus group 1 were presented with the results from the previous round of workshops. The results are summarized in Figures S2 and S3 in Multimedia Appendix 2.

## Results

### Overview

A total of 17 participants joined 3 focus groups. The demographic characteristics of participants are reported in Table 1. A total of 97 people initially made contact via recruitment methods.

**Table 1:** Demographics and clinical characteristics of the participants (N=17).

Demographic and clinical characteristics		Participants, n (%)
**Age group (y)**		
	<18	0 (0)
	18-24	1 (6)
	25-34	4 (24)
	35-44	3 (18)
	45-55	3 (18)
	55-64	4 (24)
	>65	0 (0)
**Sex**		
	Male	5 (29)
	Female	9 (53)
	Nonbinary	1 (6)
**Category of bipolar diagnosis**		
	Bipolar 1 disorder	5 (29)
	Bipolar 2 disorder	8 (47)
	Cyclothymia	1 (6)
	Bipolar disorder not specified	1 (6)
**Years of lived experience of bipolar disorder**		
	<1	0 (0)
	1-3	2 (12)
	3-5	5 (29)
	5-7	1 (6)
	>7	7 (41)

Thematic analysis resulted in 6 themes as follows: (1) purpose of using the app, (2) desired features, (3) when to use the app, (4) risks of using the app, (5) sharing with family and friends, and (6) sharing with health care professionals. For each theme, participants described positives and negatives. Tables 2-4 report themes, subthemes, and the participant identifiers.

All the results were cross-checked by someone with lived experience of BD type 1 and over a decade of experience in PPI. Participants did not provide feedback on the findings. Coding via NVivo (Lumivero) [20] was performed by 1 researcher (LAW).

**Table 2:** Quotes related to theme 1 (purpose of using the app).

Subthemes	Quotes
Subtheme: aid not a tool to be trusted blindly	“But then I think most people would rather have someone check in and say ohh you know, your app suggests there’s been a change and then for them to say Oh no actually you know, I’m fine it’s just that this has happened that’s happened.” [ID15].
Subtheme: understanding triggers	“What I have used it for is we’ll got on. I’m I’m a bit anxious for no particular reason. Am I just being bloody minded and annoyed? But a lot of the time, it’s actually. I’ve tripled my workload. I haven’t had sleep.” [ID13]
Subtheme: improving insight	“So actually, I didn't sleep well and this happened or I did sleep well and I was fine and ohh I've been spending a lot of money doing a lot of stuff and then this happened. But also what was happening with my medication and where was I in my cycle? Where was I? What exercise was my level of exercise good at high or low or yeah, so I think it’s for your own picture and your own health and everybody’s different.” [ID11]
Subtheme: objectively judging the mood	“I think one of the positives about it for people with bipolar is that objectivity and you know the objective data that comes because it also takes a bit of that pressure off the person.” [ID12]
Subtheme: prevention	“Yes, that is fair to say and to catch it quickly, you know, I mean sort of the sooner you can pick up on it and if you've got a monitoring device that can alert you, that’s even better.” [ID8]. “Bit hyper, you’re a bit, you know, you’re talking really fast or you’re having loads of different ideas about different things and stuff, and it might be that I missed that until it’s fed back to me, reflected back to me so.” [ID9]
Subtheme: safety net and reassurance	“So something in the background. Something in the background sort of saying actually, you’re doing X, Y & Z and that’s not normal for you.” [ID5] “It’s a sense of security in a way. You know, it’s almost like having someone there constantly keeping a check on your mood, even though they’re not there virtually.” [ID8]
Subtheme: self-management	“If I’m struggling, I’m at 2. What activities could I do to boost it so I’ll go for a walk or art activities so I will go back to that book that we made and try the strategies first before I went to the doctor and got a PRN so that would be how I’d monitor it.” [ID2]
Subtheme: to compensate for having fewer people around to check in on you	“I live alone so I don’t have anybody to notice. A mood tracker would help with that.” [ID5]
Subtheme: use information to adjust medication	“Yeah, lifestyle changes. Probably tweaking my medication. It could be that because sleep deprivation is a big trigger for me and it could be that I’m lacking in sleep, so the may need to give me some sleep medication.” [ID8]

**Table 3:** Quotes related to theme 2 (desired features), 3 (when to use the app), and 4 (risks of using the app).

Themes and subthemes			Quotes
**Theme 2: desired features**			
	Subtheme: accuracy is important	“Depends how good the app is, whether it does actually perform well ’cause it could be something that is obviously not correct. In a sense, it might not necessarily me being a period of mania, it could be picking up something that is questionable.” [ID9]	
	Subtheme: the advantages of passive monitoring	“And quite often I’m not even that. I’m not that aware of. Maybe what I’ve put into it ’cause I get up, do it, and then you know and go about my business. So maybe something that’s a little bit more engaging and if it’s an app that’s working in the background more rather than you know, and then I can perhaps turn to that and realize stuff that I’ve not seen before.” [ID9]	
	Subtheme: crisis plan	“We have a plan in place like that. He’s allowed to call the doctors and the mental health team and the GP that needs to be called.” [ID17]	
	Subtheme: data and app transparency	“I think I would like to see all the individual pieces of data. I feel like if that app is collecting those data points, I kind of feel like I’m entitled to see them.” [ID10]	
	Subtheme: the ease of use	“If it’s really like clunky and not that accessible or it doesn’t feel very friendly. It’s gotta be something that fits, doesn’t it? The individual.” [ID9]	
	Subtheme: medication prompts	“When I’m not well then I’m absolutely rubbish at taking medication. So yes, prompts to take medication and stuff would probably be for me a good idea.” [ID8]	
	Subtheme: personalization	“I think that people are gonna have very different preferences and people are going to want to use this in very different ways. And I think that one size isn’t gonna fit all. And if you’re gonna do this, then you need to build in that adaptability within it.” [ID15]	
**Theme 3: when to use the app**			
	Subtheme: would monitor constantly	“I think for me it would be useful to have it kind of on in the background all the time, so that then you can kind of see those periods where you’re not 100% sure what it was like. You can go back and say oh actually I’ve had this amount of time...especially when the mood’s low you can I find that I might think, oh, I’ve only been OK for a week, but it would give you a bit more of a realistic insight to say oh actually you’ve had a few months when you’ve been okay.” [ID7]	
	Subtheme: not track all the time	“As for using it every day, I probably wouldn’t. I would probably just dip in if I felt my mood starting to get a bit lower, a bit high.” [ID14]	
	Subtheme: periods when tracking is particularly important	“I guess the idea would be that the app would learn about your behavior, so it would be able to predict when you know things might be difficult for you, or you know if certain times of the year are more difficult than being able to provide that support for you and then being able to toggle things on and off.” [ID10]	
**Theme 4: risks of using the app**			
	Subtheme: data security concerns	“I think for me anyway, because you’ve got a lot of data, I’d be a little bit worried about where that data was going if it was being shared.” [ID7]	
	Subtheme: dislike of the app when unwell	“I also got really paranoid when I was ill...so you, you know, would you get to the point where you didn’t trust the app where you thought that people were trying to get you, you know, sectioned or trying to get your money off you or, you know, trying to do some sort of nefarious activities, you know, against you at that time?” [ID 14]	
	Subtheme: feeling monitored in a negative way	“Obviously there’s a danger where you feel that you’re being monitored.” [ID13]	
	Subtheme: losing autonomy	“If the app sensed you know that things weren’t going too well, you know, in whatever kind of data point that is, then it kinda feels like my, I dunno, sense of agency is removed, or autonomy...it doesn’t feel good, it doesn’t necessarily feel like an empowering thing.” [ID10]	
	Subtheme: potential for the data to be used against you	“But it depends in certain situations, like if you got an abusive partner or something like that, you might not want them to know so.” [ID2]	
	Subtheme: the risks of oversharing data	“Massive risk is taken...Overshare...there’s a real risk the if you’ve got this sort of information you could just say to anybody any stranger: look at all this. The inhibitors aren’t there. You lose the self-control, the inner voice, when you’re high.” [ID13]	
	Subtheme: the fear of mental health services	“I’d be scared of what they’d make of that information and whether they’d make decisions behind my back about my care. That yeah, that I wouldn’t agree with.” [ID4]	

**Table 4:** Quotes related to theme 5 (sharing with family and friends) and theme 6 (sharing with health care professionals).

Themes and subthemes			Quotes
**Theme 5: sharing with family and friends**			
	Subtheme: carefully choosing the right person to share with	“So I think the ability to share with other people is is good, but as a everyone said, you need to be really careful and have the options of who you share it with.” [ID14]	
	Subtheme: facilitating conversations about relapse and care	“Yeah, I think it could create some difficult conversations, but the friend would usually be able to tell pretty quickly, especially if it’s mania they’d be able to tell just by giving me a quick phone call.” [ID4]	
	Subtheme: family and friends tracking mood	“That’s it really, with me. And of course my partner does pull me back, do you know what I mean, when he sees it running out of, do you know what I mean, running of control.” [ID3]	
**Theme 6: sharing with health care professionals**			
	Subtheme: skepticism about sharing with mental health services	“They don’t take at face value what I’m telling them and therefore they are making comments like ‘Ohh sorry, we don’t believe you’ and all this sort of stuff, so I’m opening up saying I’m ill and they're saying but you’re talking to me, you’re presenting a very strong individual, go away.” [ID13]	
	Subtheme: mood monitoring to guide treatment	“I will say it amazed me actually as in I could see my highs and my lows and I’d be like you know where as if I hadn’t been doing that, I wouldn’t have been able to put that across to the health professional because it would kind of just gone you know, so this...was actually really beneficial to me.” [ID16]	
	Subtheme: sharing less with professionals versus family and friends	“Do it myself first and share that sort of more directly with all my husband or whatever rather than sharing it sort of with the mental health team straight off.” [ID7]	
	Subtheme: sharing relies on trust	“I think you’d have to be at one with who you’re sharing with so you wouldn’t just be sharing indiscriminately with health care professionals who that don’t know you...my consultant psychiatrist, I’ve had, you know for 16, almost 16 years, so you know, it's lucky for me that I know her and true her and she knows me.” [ID12]	
	Subtheme: would share in retrospect but not in real time	“Retrospectively, like it would work, I guess in the same way how I've been like using like the previous apps, I think in real time, I guess it could be like quite a few problems.” [ID10]	

Participants articulated a wide variety of different needs and preferences for the use of the app; some were commonly endorsed, and some were only endorsed by a few but for specific reasons, for example, because they lived alone and needed more objectivity (Tables 3 and 4). Most (35/45, 78%) subthemes were articulated, even if very briefly, in all 3 focus groups. Naturally, some subthemes were discussed in much more depth in certain focus groups. This is likely to represent important and generalizable data [21]. A minority (13/45, 29%) of subthemes were present in 1 or 2 focus groups (eg, medication prompts, providing continuity of care, positive messages, reassurance, data security concerns, feeling monitored [negative], potential for abuse, the risks of oversharing data, facilitating conversations about relapse and care, skepticism about sharing with mental health services, monitoring constantly, self-rating of mood can be unreliable, and stop self-monitoring when unwell).

### Theme 1: Purpose of Using the App

Views on the purpose of the app were mixed (Table 3), and the participants wished to use the app in a variety of different ways. Some participants reported multiple uses for the app; others wished to use it for one specific purpose. The most popular was using the information they inputted into and reflecting back from the app to get a sense of where their mood was at that moment and to self-manage their BD. This could take the form of scaling back commitments, communicating with one’s support network, changing medication, ensuring sufficient sleep, and doing more exercise, depending on the mood state. Many people wished to share information about their mood with trusted individuals around them and some with their health care professional.

Many wished to use the app to prevent future relapse by identifying early warning signs and triggers and improving long-term insight by examining both where their mood was now and the long-term changes in mood over many months and years. Many wanted a safety net for when they lost insights into how low or high their mood was at that moment, to both prevent the worsening of their mood and to avoid the negative behavioral consequences of being unwell, for example, excessive spending leading to the accumulation of long-term debt. There was an acknowledgment that the app might occasionally be wrong and that this information would be used to inform decision-making but not be the sole driver of it.

### Theme 2: Desired Features

Participants were complimentary of passive monitoring (provided it was accurate and gave usable data), particularly regarding the ease of tracking their data over many years and when they may not wish to do so, for example, when they were unwell (Table 4). Many identified that a key barrier to them tracking their mood at present was the amount of time and effort it took, which they felt passive approaches would solve. Function and usability were key—the app being accurate in its core functions, for example, roughly identifying mood state. Participants wished to incorporate a crisis plan and personal alert system into the app; suggestions included writing things a person wished to be reminded of when they were well (such as a crisis plan) that could then be shown to them when they were unwell, for example, around self-management of the illness. Another suggestion was receiving a notification from the app simply informing a person that they might be unwell as well as potentially sharing that with a predetermined trusted individual (explored in more detail in Theme 5: Sharing With Family and Friends section). The ability to customize and personalize aspects of the app seemed key, for example, toggling on-off certain aspects of data collection or any sharing. It was acknowledged that individuals would wish to use the app in a variety of different ways with different preferences around data security and the way they manage their BD.

### Theme 3: When to Use the App

Opinions regarding when participants might use the app were mixed (Table 4). Some wished to monitor it constantly, acknowledging that doing so may help them triangulate their mood around important life events in the hope of managing their illness. Others disliked the idea of tracking all the time and did not wish to do so when well. Some mentioned that there were times, for example, around particularly stressful life events, when tracking might be more important.

### Theme 4: Risks of Using the App

Concerns were raised around data security and possible breaches of data (Table 4), including from the individual who was unwell, for example, oversharing their information or data with someone who could potentially harm them. Susceptibility to abuse because of the collected data were a concern and some participants felt as the stigma of their illness had been used against them previously. Some participants were concerned they might feel monitored and that they would develop a dislike of the app if they felt particularly paranoid when unwell, potentially deleting it or removing their data from it. In total, 2 participants expressed concerns that the app could decrease their sense of autonomy, particularly if it was directing a person to do certain things or sharing their information with others. Many expressed concerns and fear of the involvement of mental health services deriving from previous negative experiences, including not feeling heard, being detained under the Mental Health Act, and a lack of trust in their health care professional.

### Theme 5: Sharing With Family and Friends

We explored sharing with others in detail as it appeared to be a particularly important and potentially difficult-to-implement aspect of the app (Table 4). Participants felt that carefully choosing the right person to share any data with was extremely important. Some participants felt that their family and friends would wish to see their mood charts over long periods, while others felt that that information would not be of interest. Some wished for a single chosen person or a couple of chosen people to receive a notification when they were unwell as a safety net—building on previous nuanced conversations around safety or crisis planning, relapse, self-management, the involvement of mental health services, etc. Others would not choose this functionality and would not wish to share their data.

### Theme 6: Sharing With Health Care Professionals

Many individuals did not wish to share their mood data with their health care professional in real time, due to concerns about an overreaction from mental health services, coercion, and a preference for self-management (Table 4). This seemed to be determined by the degree of trust participants had in their health care professional. However, most would share the data about their mood in retrospect to help with diagnosis, assessment, and subsequent treatment, for example, medication monitoring.

## Discussion

### Principal Findings

This qualitative study explored what people with BD wanted from a passive, mood-tracking app and tool to help them manage their BD. We explored the purpose, desired features, and usability in the context of ethical development of technology considering potential risks and safety of use and misuse. We explored the potential methods of incorporating desired features into the app in ways that would not compromise safety or cause unintended consequences, for example, sharing data with others.

Fundamentally, people with BD wanted to use passive mood-monitoring technology for a wide variety of purposes using different methods. They wished to use the data in a diverse and pragmatic way to help them self-manage their BD with greater insight and a better understanding of potential triggers. We discuss key features that may aid this functionality, for example, sharing with trusted individuals and reminders of crisis plans or personal alert systems. We discuss the future development of passive mood-monitoring technologies and particularly state that it should not be assumed that the involvement of formal mental health services is desired.

Most individuals principally wished to use the app to aid the already existing self-management of their BD. The way individuals wished to self-manage varied, and a preference for personalization was clear to accommodate the variety of strategies of using the app and the data it generated. This accords with previous work where insight, understanding, and self-management [6] to aid coping and prevention of relapse were the most salient reasons for engaging with self-monitoring [5,22-24]. Some subthemes were only articulated by a minority of participants, for example, using the information from the app to adjust their medication, providing continuity of care, and receiving positive or encouraging messages from the app. These less frequently endorsed subthemes highlight the multiple, different, and individual ways that the data generated by the app could be used. This kind of technological intervention could present data to individuals who can then use these data flexibly to prompt a variety of more nuanced behavioral changes.

Sharing with health care professionals appeared to be a less important reason for developing the app; again, this chimes with previous findings [5]. Individuals in this study felt that sharing their data and facilitating conversations around their illness with someone and people they trust was important, reinforcing what previous findings had demonstrated [5]. Communication with trusted people to improve relationships and manage the condition together seems important for many people with BD, while for others who do not have a trusted individual to check on them, the app seemed to provide an alternative safety net.

Fundamentally the app needed to be easy to use and accurate when determining mood. This presents programming and technical challenges around performance, validity, and human-computer interaction. The RADAR platform, as used in depression, multiple sclerosis, and epilepsy, uses passive monitoring to obtain a general detection of important changes in the underlying condition, which then triggers active data completion to provide more granularity and greater sensitivity and specificity [17,25,26]. Participants articulated that the app does not necessarily have to be accurate all the time (subtheme: aid not a tool to be trusted blindly) and that any data from the app would be triangulated with contextual information, such as their own personal judgment on their mood and the opinions of those around them. It does seem important to have an awareness of how accurate the app is likely to be and this will factor into any decision-making that is reliant on the app’s data [27].

Participants in our study suggested feasible ways of incorporating desired features and mitigating the risks of this technology. Crisis plans and personal alert systems could be set up when an individual is well, and then they could be reminded of crisis plans and personal alert systems when they are unwell (similar methods have been used in diabetes care [28] and epilepsy [29]). The person with BD could nominate 1 individual to receive a notification if the app considered that the person with BD might be unwell—again, similar to recent developments with seizure or diabetes tracking apps [28,29]. These features can all be chosen actively and toggled on and off, including all individual data collection methods. This would incorporate some aspects of personalization that seem important to people with BD, both in this study and in others, to account for differing needs, patterns of illness, and self-management strategies [5,22,24,30]. Data security should be considered carefully, and we should assume that many individuals will be skeptical about sharing their data with mental health services.

Participants in our focus groups did not feel that the app would feel confronting and potentially exacerbate any negative thoughts [31] via increasing awareness of depressive symptoms; however, other studies have suggested that this may be the case with mood-monitoring apps [32,33]. Some participants were concerned about the risks of using the app and there was a concern about technology exacerbating the risks that people with BD already face, for example, vulnerability, abuse, a loss of autonomy and independence, and stigma.

### Limitations

The limitations of the study are mentioned subsequently. The sample reported here is small and is unlikely to be representative of those with BD more broadly, partly because we specifically recruited individuals with an interest in smartphone-based interventions and mood tracking. However, it did show diversity, both in terms of demographics and subthemes and views articulated. The recruitment was based on the kind of people we expected would ultimately use the app therefore was a pragmatic sampling method to best inform the final intervention. As most of these individuals were interested in mood-monitoring interventions, the degree of engagement and detailed discussion was likely higher than if we had selected individuals who did not have a particular interest in mood-tracking or digital interventions. Another limitation of this work was that we did not systematically record ethnicity data or previous experience with mood-tracking platforms. We were limited to how much personal data we collected and reported so that the data, combined with direction quotations, would not identify participants who were assured of anonymity through ethical review and individual consent.

The app was discussed hypothetically with a demonstration or discussion of possible features or usability; therefore, participants did not use the app. It is possible that following use, opinions may change; however, mostly the discussion around potential features, risks, and usability was theoretical, hence we decided to include a hypothetical discussion. Further work could iteratively develop the app, incorporating this feedback and these views into its development. A series of rounds of qualitative work could be conducted to gradually develop a feasible, usable, and acceptable app for people with BD. Furthermore, these results are specific to the RADAR platform, but there are many commonalities between passive behavior-tracking apps. Therefore, it is reasonable to consider the applicability of other platforms. These focus groups highlight the importance of accuracy and performance of digital mood-tracking apps [32,34,35].

Using the principles of RRI, we have considered and reflected on the issues identified by stakeholders as well as mitigation practices in the iterative development of this app; however, this is largely hypothetical. As we develop the app further into a useable prototype and the clinical utility is increasingly explored, we will continue engaging with stakeholders to anticipate and act upon risk detection and risk surveillance to anticipate potential misuse or unintended use as well as prioritizing safety, inclusivity, and responsible innovation [11]. This is particularly important considering the potential risks or harms highlighted here and previous concerns around the potential adverse effects of mood-monitoring interventions [32,33].

### Conclusions

This study suggests that people with BD, who are interested in using passive technology to monitor mood, wish to do so for a wide variety of purposes and using a variety of different methods of using the data. Principally, people with BD wished to use this novel technology to aid them in self-managing their BD with greater insight and a better understanding of potential triggers. There are a few key features that may aid this functionality, for example, sharing with trusted individuals and the reminder of crisis plans and personal alert systems. Future development of passive mood-monitoring technologies should not assume that the involvement of formal mental health services is desired.

## Appendix

Multimedia Appendix 1 COREQ (Consolidated Criteria for Reporting Qualitative Studies) checklist.

Multimedia Appendix 2 Details of possible data collection and summary of previous works.

## Abbreviations

BD: bipolar disorder
COREQ: Consolidated Criteria for Reporting Qualitative Studies
PPI: patient and public involvement
RADAR: Remote Assessment of Disease and Relapse
RRI: responsible research and innovation

## Data Availability

The datasets generated during and analyzed during this study are available from the corresponding author on reasonable request.

## References

[1] Torous J, Bucci S, Bell IH, Kessing LV, Faurholt-Jepsen M, Whelan P, Carvalho AF, Keshavan M, Linardon J, Firth J (2021). The growing field of digital psychiatry: current evidence and the future of apps, social media, chatbots, and virtual reality. World Psychiatry.

[2] Torous J, Choudhury T, Barnett I, Keshavan M, Kane J (2020). Smartphone relapse prediction in serious mental illness: a pathway towards personalized preventive care. World Psychiatry.

[3] Depp C, Torous J, Thompson W (2016). Technology-based early warning systems for bipolar disorder: a conceptual framework. JMIR Ment Health.

[4] Matcham F, Barattieri di San Pietro C, Bulgari V, de Girolamo G, Dobson R, Eriksson H, Folarin AA, Haro JM, Kerz M, Lamers F, Li Q, Manyakov NV, Mohr DC, Myin-Germeys I, Narayan V, Bwjh P, Ranjan Y, Rashid Z, Rintala A, Siddi S, Simblett SK, Wykes T, Hotopf M (2019). Remote assessment of disease and relapse in major depressive disorder (RADAR-MDD): a multi-centre prospective cohort study protocol. BMC Psychiatry.

[5] Majid S, Morriss R, Figueredo G, Reeves S (2022). Exploring self-tracking practices for those with lived experience of bipolar disorder: learning from combined principles of patient and public involvement and HCI. Proceedings of the 2022 ACM Designing Interactive Systems Conference.

[6] Murnane E, Cosley D, Chang P, Guha S, Frank E, Gay G, Matthews M (2016). Self-monitoring practices, attitudes, and needs of individuals with bipolar disorder: implications for the design of technologies to manage mental health. J Am Med Inform Assoc.

[7] Morriss R, Vinjamuri I, Faizal MA, Bolton CA, McCarthy JP (2013). Training to recognise the early signs of recurrence in schizophrenia. Cochrane Database Syst Rev.

[8] Bauer M, Glenn T, Alda M, Grof P, Bauer R, Ebner-Priemer UW, Ehrlich S, Pfennig A, Pilhatsch M, Rasgon N, Whybrow PC (2023). Longitudinal digital mood charting in bipolar disorder: experiences with chronorecord over 20 years. Pharmacopsychiatry.

[9] Post RM, Denicoff KD, Leverich GS, Altshuler LL, Frye MA, Suppes TM, Rush AJ, Keck PE, McElroy SL, Luckenbaugh DA, Pollio C, Kupka R, Nolen WA (2003). Morbidity in 258 bipolar outpatients followed for 1 year with daily prospective ratings on the NIMH life chart method. J Clin Psychiatry.

[10] Bos F, Schreuder M, Doornbos B, Snippe E, Bruggeman R, van der Krieke L, Haarman B, Wichers M, George S (2021). Prospective early warning signals to detect transitions to manic and depressive episodes in bipolar disorder. Eur Psychiatry.

[11] Eyre HA, Ellsworth W, Fu E, Manji HK, Berk M (2020). Responsible innovation in technology for mental health care. Lancet Psychiatry.

[12] Stilgoe J, Owen R, Macnaghten P (2013). Developing a framework for responsible innovation. Res Policy.

[13] Jirotka M, Grimpe B, Stahl B, Eden G, Hartswood M (2017). Responsible research and innovation in the digital age. Commun ACM.

[14] Lopez-Campos G, Gabarron E, Martin-Sanchez F, Merolli M, Petersen C, Denecke K (2024). Digital interventions and their unexpected outcomes - time for digitalovigilance?. Stud Health Technol Inform.

[15] Middle R, Welch L (2022). Experiences of digital exclusion and the impact on health in people living with severe mental illness. Front Digit Health.

[16] Greer B, Robotham D, Simblett S, Curtis H, Griffiths H, Wykes T (2019). Digital exclusion among mental health service users: qualitative investigation. J Med Internet Res.

[17] Sun S, Folarin AA, Zhang Y, Cummins N, Liu S, Stewart C, Ranjan Y, Rashid Z, Conde P, Laiou P, Sankesara H, Dalla Costa G, Leocani L, Sørensen PS, Magyari M, Guerrero AI, Zabalza A, Vairavan S, Bailon R, Simblett S, Myin-Germeys I, Rintala A, Wykes T, Narayan VA, Hotopf M, Comi G, Dobson RJ (2022). The utility of wearable devices in assessing ambulatory impairments of people with multiple sclerosis in free-living conditions. Comput Methods Programs Biomed.

[18] Simblett S, Matcham F, Curtis H, Greer B, Polhemus A, Novák J, Ferrao J, Gamble P, Hotopf M, Narayan V, Wykes T (2020). Patients' measurement priorities for remote measurement technologies to aid chronic health conditions: qualitative analysis. JMIR Mhealth Uhealth.

[19] Polhemus A, Novak J, Majid S, Simblett S, Morris D, Bruce S, Burke P, Dockendorf MF, Temesi G, Wykes T (2022). Data visualization for chronic neurological and mental health condition self-management: systematic review of user perspectives. JMIR Ment Health.

[20] Castleberry A (2014). NVivo 10 [software program]. Version 10. QSR international; 2012. Am J Pharm Educ.

[21] Guba EG (1981). Criteria for assessing the trustworthiness of naturalistic inquiries. Educ Technol Res Dev.

[22] Bos FM, Snippe E, Bruggeman R, Doornbos B, Wichers M, van der Krieke L (2020). Recommendations for the use of long-term experience sampling in bipolar disorder care: a qualitative study of patient and clinician experiences. Int J Bipolar Disord.

[23] Saunders KE, Bilderbeck AC, Panchal P, Atkinson LZ, Geddes JR, Goodwin GM (2017). Experiences of remote mood and activity monitoring in bipolar disorder: a qualitative study. Eur Psychiatry.

[24] Bos FM, Snippe E, Bruggeman R, Wichers M, van der Krieke L (2019). Insights of patients and clinicians on the promise of the experience sampling method for psychiatric care. Psychiatr Serv.

[25] Matcham F, Leightley D, Siddi S, Lamers F, White KM, Annas P, de Girolamo G, Difrancesco S, Haro JM, Horsfall M, Ivan A, Lavelle G, Li Q, Lombardini F, Mohr DC, Narayan VA, Oetzmann C, Penninx BW, Bruce S, Nica R, Simblett SK, Wykes T, Brasen JC, Myin-Germeys I, Rintala A, Conde P, Dobson RJ, Folarin AA, Stewart C, Ranjan Y, Rashid Z, Cummins N, Manyakov NV, Vairavan S, Hotopf M (2022). Remote Assessment of Disease and Relapse in Major Depressive Disorder (RADAR-MDD): recruitment, retention, and data availability in a longitudinal remote measurement study. BMC Psychiatry.

[26] Simblett SK, Biondi A, Bruno E, Ballard D, Stoneman A, Lees S, Richardson MP, Wykes T (2020). Patients' experience of wearing multimodal sensor devices intended to detect epileptic seizures: a qualitative analysis. Epilepsy Behav.

[27] Andrews JA, Craven MP, Jamnadas-Khoda J, Lang AR, Morriss R, Hollis C, Consortium R (2020). Health care professionals' views on using remote measurement technology in managing central nervous system disorders: qualitative interview study. J Med Internet Res.

[28] Debong F, Mayer H, Kober J (2019). Real-world assessments of mySugr mobile health app. Diabetes Technol Ther.

[29] Alzamanan MZ, Lim KS, Akmar Ismail M, Abdul Ghani N (2021). Self-management apps for people with epilepsy: systematic analysis. JMIR Mhealth Uhealth.

[30] Majid S, Reeves S, Figueredo G, Brown S, Lang A, Moore M, Morriss R (2021). The extent of user involvement in the design of self-tracking technology for bipolar disorder: literature review. JMIR Ment Health.

[31] van Genugten CR, Schuurmans J, Lamers F, Riese H, Penninx BW, Schoevers RA, Riper HM, Smit JH (2020). Experienced burden of and adherence to smartphone-based ecological momentary assessment in persons with affective disorders. J Clin Med.

[32] Dubad M, Winsper C, Meyer C, Livanou M, Marwaha S (2018). A systematic review of the psychometric properties, usability and clinical impacts of mobile mood-monitoring applications in young people. Psychol Med.

[33] Palmier-Claus J, Lobban F, Mansell W, Jones S, Tyler E, Lodge C, Bowe S, Dodd A, Wright K (2021). Mood monitoring in bipolar disorder: is it always helpful?. Bipolar Disord.

[34] Faurholt-Jepsen M, Munkholm K, Frost M, Bardram JE, Kessing LV (2016). Electronic self-monitoring of mood using IT platforms in adult patients with bipolar disorder: a systematic review of the validity and evidence. BMC Psychiatry.

[35] Ortiz A, Maslej MM, Husain MI, Daskalakis ZJ, Mulsant BH (2021). Apps and gaps in bipolar disorder: a systematic review on electronic monitoring for episode prediction. J Affect Disord.

