# Supplementary material for: Improving the Utility, Safety, and Ethical Use of a Passive Mood-Tracking App for People With Bipolar Disorder Using Coproduction: Qualitative Focus Group Study
Source: JMIR Form Res. 2025 Feb 7;9:e65140. doi: 10.2196/65140 (PMC11845880; doi:10.2196/65140)

Supplementary Figure 1: Potential data collection via the RADAR passive behavioural tracking app.


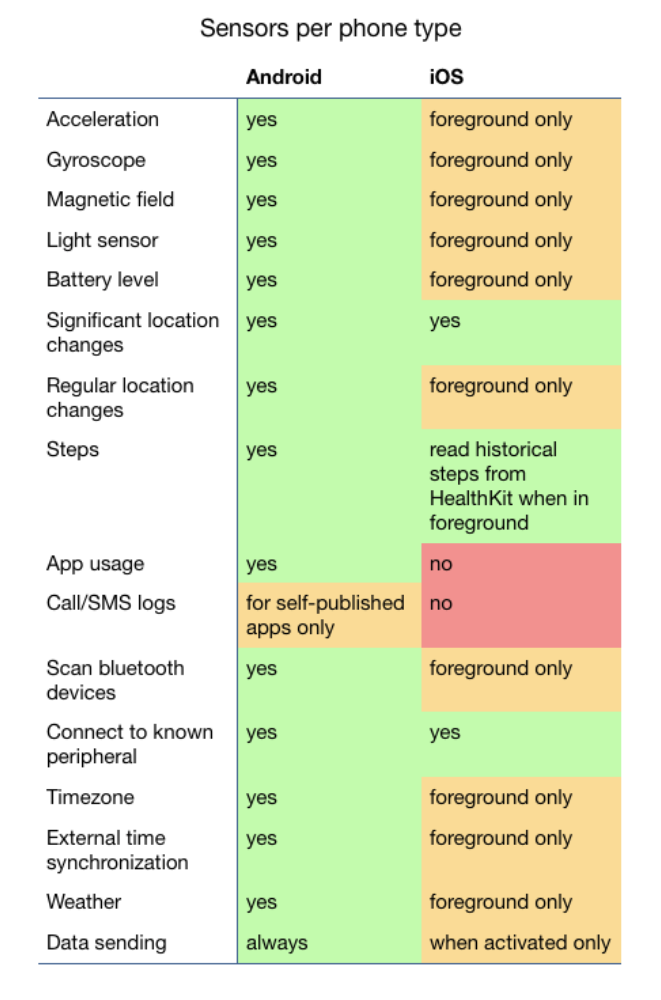


Supplementary Figure 2: Summary of results from previous round of workshops exploring motivations for general mood tracking as well as the utility of this


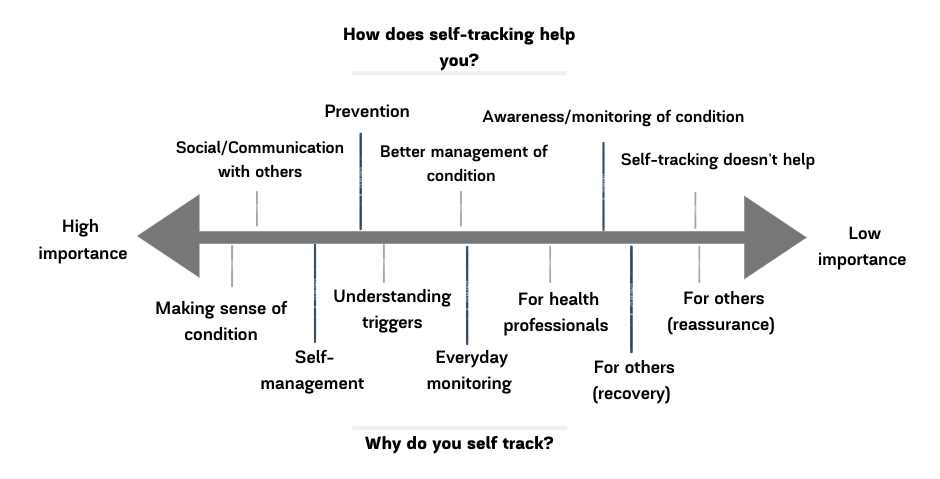


Supplementary Figure 3: Summary of results from previous round of workshops exploring the potential benefits of mood tracking


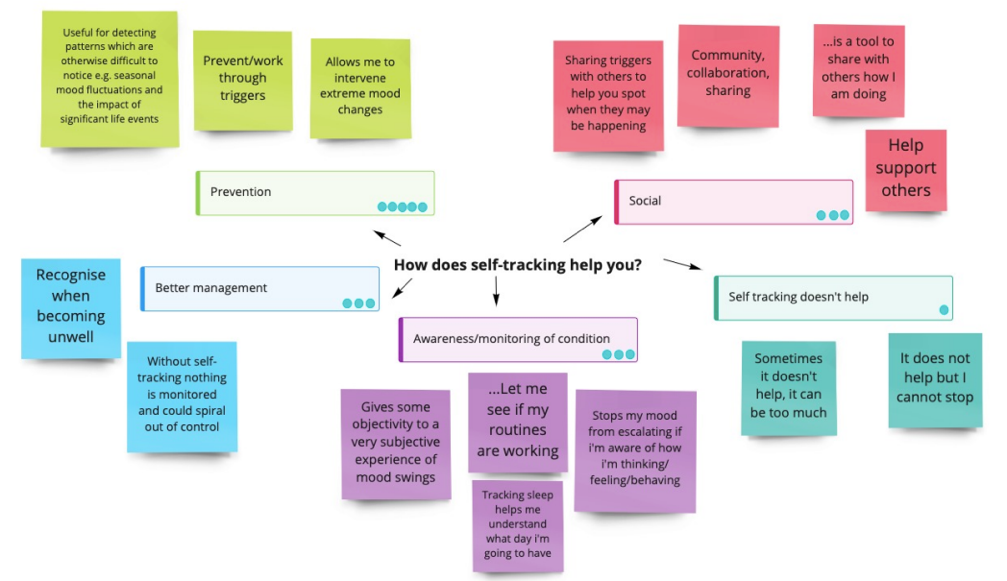

Supplement: Multimedia Appendix 2 [file formative_v9i1e65140_app2.docx]
